# Charge‐Transfer‐Mediated Boron Magneto‐Ionics: Towards Voltage‐Driven Multi‐Ion Transport

**DOI:** 10.1002/advs.76552

**Published:** 2026-07-11

**Authors:** Zheng Ma, Karim‐Alexandros Kantre, Huan Tan, Aitor Arredondo‐López, Maciej O. Liedke, Javier Herrero‐Martín, Eric Hirschmann, Andreas Wagner, Daniel Mora‐Blanco, Alberto Quintana, Salvador Pané, Eva Pellicer, Josep Nogués, Johan Meersschaut, Jordi Sort, Enric Menéndez

**Affiliations:** ^1^ Departament de Física Universitat Autònoma de Barcelona Cerdanyola del Vallès Spain; ^2^ IMEC Leuven Belgium; ^3^ Institute of Radiation Physics Helmholtz‐Zentrum Dresden – Rossendorf Dresden Germany; ^4^ ALBA Synchrotron Light Facility Barcelona Spain; ^5^ Multi‐Scale Robotics Laboratory Institute of Robotics and Intelligent Systems ETH Zurich Zurich Switzerland; ^6^ Catalan Institute of Nanoscience and Nanotechnology (ICN2) CSIC and BIST Barcelona Spain; ^7^ Institució Catalana de Recerca i Estudis Avançats (ICREA) Barcelona Spain

**Keywords:** charge transfer, coercivity, magneto‐ionics, multi‐ion transport, squareness, voltage control of magnetism

## Abstract

Voltage control of magnetism via magneto‐ionics, where ion transport and/or redox processes drive magnetic modulation, holds great promise for next‐generation memories and computing. This stems from its non‐volatility and ability to precisely tune both the magnitude and speed of magnetic properties in a potential energy‐efficient manner. However, expanding magneto‐ionics to incorporate novel mobile ions or even multiple ion species is crucial for unlocking new phenomena and enabling multifunctional capabilities. Here, we demonstrate voltage‐driven multi‐ion transport in an FeBO system with increasing oxygen content, progressively transitioning from an electrostatic‐like response to a more pronounced electrochemical (magneto‐ionic) behavior. The voltage‐driven transport of both B and Fe is activated by oxidation state tuning, owing to the larger electronegativity of oxygen. Such charge‐transfer effects allow multi‐ion magneto‐ionics, where O ions move oppositely to Fe and B ions. These results pave the way for programmable functionalities by leveraging elements with different electron affinities through charge‐transfer engineering.

## Graphical abstract

This study demonstrates voltage‐driven multi‐ion transport in FeBO systems, revealing simultaneous motion of B, Fe, and O ions enabled by charge‐transfer effects. By tuning oxidation states through oxygen incorporation, we achieve a transition from electrostatic‐like to electrochemical magneto‐ionic behavior. The findings establish boron magneto‐ionics, advancing programmable, energy‐efficient control of magnetic properties for next‐generation multifunctional spintronic applications.

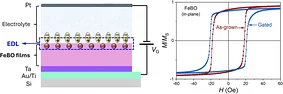

## 1 Introduction

The use of voltage to control magnetism through converse magneto‐electric effects has emerged as a promising approach for developing energy‐efficient spintronic devices. Among the different magneto‐electric mechanisms, magneto‐ionics, where voltage‐driven ion transport and/or redox processes modulate magnetism, stands out due to its non‐volatility and versatility in modifying the magnitude and speed of magnetic response [1]. So far, magneto‐ionic systems/devices have primarily relied on single‐ion manipulation, such as H^+^ [2], Li^+^ [3], O^2−^ [4, 5], F^−^ [6], OH^−^ [7], or N^3−^ [8], or C^x−^ [9]. Even though dual‐type magneto‐ionics has been demonstrated [10, 11, 12], expanding the range of mobile ions and enabling multi‐ion transport are pivotal for achieving integrated multifunctionality and on‐demand programmable magneto‐ionics. By moving beyond a single redox channel, multi‐ion transport introduces additional degrees of freedom that allow more versatile and potentially decoupled magnetic state engineering, thereby enriching functional tunability. Through the coordinated involvement of chemically distinct species with differentiated activation regimes, multi‐ion magneto‐ionics offers advantages for voltage control of magnetism toward a chemically programmable and multifunctional materials platform, eventually suitable for advanced plasticity (and metaplasticity) memory effects [13].

Here we report, to our knowledge, the first demonstration of voltage‐driven B‐ion migration in FeBO‐based heterostructures, enabled by engineering the oxidation state of B through oxygen incorporation. The presence of oxygen has a synergetic two‐fold effect: (i) it alters the oxidation state of B in FeBO heterostructures, shifting it toward a more positive value as the oxygen‐to‐argon pressure ratio increases during sputtering, an effect driven by oxygen's higher electronegativity, and (ii) it increases resistivity, enhancing electric field penetration and facilitating voltage‐driven ion transport [14]. In FeB, ion diffusion is mainly restricted to the top layers due to surface passivation. The system is weakly magneto‐ionic, showing an electrostatic‐like response [15]. However, as the oxygen content in FeBO heterostructures increases, the electrochemical (magneto‐ionic) response is enhanced due to the increased resistivity, with oxygen ions migrating in the opposite direction to Fe and B ions. Namely, an unprecedented voltage‐induced multi‐ion magneto‐ionic mechanism is observed, which not only alters the magnetization but also tends to increase coercivity upon voltage actuation.

## 2 Results and Discussion

50 nm‐thick FeBO films were sputtered at room temperature on Si(100) wafers, previously coated with 15 nm Ti, 50 nm Au, and 10 nm Ta, under varying O_2_/Ar gas flow ratios (i.e., $\frac{O_{2}\;flow}{O_{2}\;flow+\mathrm{Ar}\;flow}$) — 0 (no oxygen), 2, and 5% O_2_, denoted hereafter as FeB, FeBO (2% O_2_), and FeBO (5% O_2_), respectively (Methods). The microstructure of the FeB and FeBO (2% O_2_) is quite different. FeB presents an amorphous‐like homogeneous morphology (Figure S1), while FeBO (2% O_2_) shows a columnar‐shaped structure with elongated grains of about 3 nm in diameter (Figure 1a), triggered by the presence of oxygen [16]. Moreover, the high‐angle annular dark‐field scanning transmission electron microscopy (HAADF‐STEM) and elemental electron energy loss spectroscopy (EELS) mappings of the FeBO (2% O_2_) sample (Figure 1b) show that, apart from a top overoxidized 2–3 nm layer (due to exposure to air), the Fe and O are homogeneously distributed throughout the film. The B signal, which is challenging to measure using EELS, is at the background level and is thus not shown. The fast Fourier transform (FFT) pattern in Figure 1c shows predominantly a diffuse diffraction ring and a hallo, evidencing the highly nanostructured nature of the film, together with some diffraction spots, all compatible with a phase close to metallic Fe (PDF 00‐001‐1262). This is consistent with the lattice fringes detected in sporadic regions of the as‐grown FeBO (2% O_2_) sample (see Figure S2). Grazing‐incidence x‐ray diffraction measurements of the as‐grown films (Figure S3) exhibit only very weak diffraction features, indicative of a predominantly nanostructured character. A faint peak at 65°–66° is compatible with body‐centered cubic Fe (mp‐13) and Ti (mp‐72), and can therefore be associated with the FeB layer and/or the Ti buffer layer. Additional traces near 37° and 56° can be attributed to Au (mp‐81) and Si (mp‐149), respectively [17].

**FIGURE 1:**
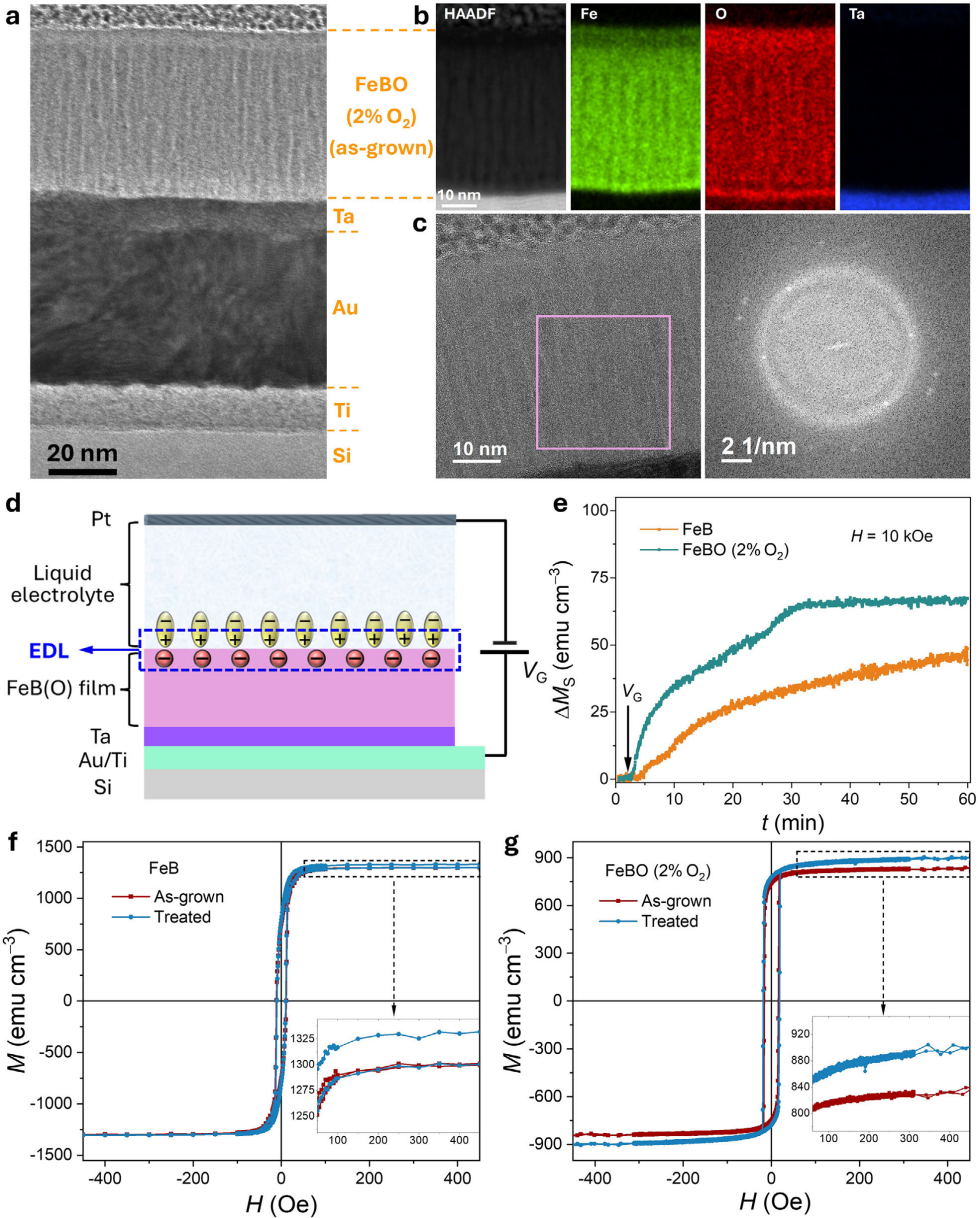
Voltage control of magnetism in FeB‐ and FeBO‐based heterostructures. (a) Cross‐sectional TEM image of the as‐grown FeBO (2% O_2_) heterostructure, showing the columnar growth of the film. (b) HAADF‐STEM micrograph of the heterostructure and the corresponding Fe, O, and Ta elemental EELS mappings. (c) High‐resolution TEM image of the FeBO layer, together with the FFT pattern of the region enclosed by the red square. (d) Schematic of the sample and electrolyte gating configuration used. As a consequence of biasing, an electric double layer (EDL) forms at the film/electrolyte interfaces. (e) Variation of saturation magnetization (i.e., Δ*M*_S_) as a function of time *t* while applying voltage. Note that the curves were recorded while an in‐plane magnetic field of 10 kOe was applied. Gate voltages, *V*_G_, were applied starting from *t*  =  2 min. (f,g) Comparison of the hysteresis loops of the as‐grown and gated (−50 V for 1 h) FeB and FeBO (2% O_2_) samples, respectively. (f) Hysteresis loops of the as‐grown and gated (−50 V for 1 h) FeB films. (g) Hysteresis loops of the as‐grown and gated (−50 V for 1 h) FeBO (2% O_2_) films.

To investigate magneto‐electric effects of the different samples, room‐temperature magnetometry measurements were carried out while electrolyte gating the heterostructures (see Figure 1d and Methods). Figure 1e displays the evolution of the change in saturation magnetization, Δ*M*_S_, while applying a gate voltage of −50 V, for the FeB and FeBO (2% O_2_) heterostructures. Both films exhibit ferromagnetism in the as‐grown state (Figure 1f,g) [18], and their Δ*M*_S_ increase as a response to biasing. Subsequently, after gating for 1 h, the voltage was turned off, and hysteresis loops were recorded. As shown in Figure 1g, Supporting Information, the increase in saturation magnetization (*M*_S_) is permanent (non‐volatile) for the FeBO (2% O_2_) sample, compatible with the partial reduction of FeBO to metallic FeB (i.e., magneto‐ionics). In contrast, the voltage‐induced *M*_S_ increase in the FeB sample is less pronounced and shows volatility, recovering the initial value of the as‐grown sample during the duration of the hysteresis loop (Figure 1f), indicating that FeB exhibits minimal magneto‐ionic behavior.

To gain insight into the magnetic anisotropy of the as‐grown FeB and FeBO (2% O_2_) films, in‐plane angular‐dependent *M*‐*H* measurements were performed (Figures S4 and S5). The as‐grown FeB sample (Figure S4a,c,d) exhibits clear in‐plane uniaxial magnetic anisotropy, evidenced by the two‐fold symmetry (180° periodicity) of the squareness ratio (*M*_R_/*M*_S_), revealing well‐defined magnetic easy and hard axes. The angular dependence of coercivity (*H*_C_) also suggests uniaxial anisotropy, although less clearly, since coercivity is more strongly influenced by local reversal processes such as domain‐wall nucleation and pinning. Consequently, *M*_R_/*M*_S_ often provides a more reliable fingerprint of the easy‐axis direction [19, 20].

After gating (Figure S4b–d), the uniaxial anisotropy becomes equally evident in both *M*_R_/*M*_S_ and *H*_C_ angular dependences, implying voltage‐induced microstructural modifications. Since the magneto‐ionic response is weak and volatile (Figure 1f), these modifications are likely confined to the near‐surface region. Considering the amorphous‐like or weakly crystalline nature of the films (Figures S1–S3), the anisotropy is unlikely to originate from conventional magnetocrystalline effects [20, 21]. Instead, it is more plausibly associated with deposition‐induced symmetry breaking, including bond‐orientational anisotropy, anisotropic residual stresses, and interface‐induced effects [21, 22, 23]. Consistently, the treated FeB heterostructure (Figure S4b) exhibits double contributions in the 105° loop and recoil‐curve overshoots in the 30° loop, indicating the coexistence of magnetic regions with slightly different properties, likely associated with small misalignments of the magnetic easy axes. These observations support that even the weak voltage‐driven effects are sufficient to induce subtle microstructural modifications. Voltage actuation in electrostatic‐responsive materials, such as metallic thin films like FePt [24], and porous Cu‐Ni [25], as well as in systems displaying mixed electrostatic/electrochemical behavior [26], generally leads to a reduction in *H*_C_. In electrostatic systems, this is usually attributed to voltage‐induced modifications in electronic density, whereas in mixed systems, it is mainly linked to magneto‐ionic effects, where ion transport and redox processes increase the ferromagnetic fraction, enhance percolation, and strengthen exchange interactions, ultimately reducing coercivity [27]. In contrast, our results reveal an opposite trend, indicating that uniaxial anisotropy introduces an additional competing energy term that governs the coercivity evolution under voltage.

Figure S5 presents the in‐plane angular‐dependent *M*‐*H* measurements for the as‐grown and treated FeBO (2% O_2_) heterostructures. In contrast to FeB, both samples exhibit a much more isotropic magnetic behavior, with only traces of uniaxial magnetic anisotropy remaining in the angular dependence of the coercivity. The absence of a clear two‐fold symmetry in *M*_R_/*M*_S_ indicates strongly weakened anisotropy, with residual anisotropic contributions remaining detectable only in *H*_C_ due to its sensitivity to local magnetization reversal processes. This behavior highlights the role of oxygen incorporation in weakening the uniaxial magnetic anisotropy through modifications of the local Fe‐B/Fe‐Fe coordination caused by the formation of additional B‐O and Fe─O bonds, changes in residual stress, and alterations in the magnetic connectivity of the Fe‐rich matrix. These observations suggest that oxygen‐driven changes in the oxidation state of B and Fe play a central role in determining the magnetic properties. Nevertheless, despite the more isotropic character, the voltage‐induced evolution of *H*_C_ remains opposite to that typically observed in oxide‐based systems with mixed responses, showing an increase in coercivity for most angles upon gating, suggesting that B may play a role in this unusual magneto‐electric response of FeBO (2% O_2_).

To further clarify the role of B, an FeO (2% O_2_) control film without B was electrolyte‐gated, showing the expected reduction in coercivity (Figure S6). This result highlights the key role of B in the unusual magneto‐electric response of FeBO (2% O_2_).

Magnetic anisotropy was further characterized by performing in‐plane to out‐of‐plane angular‐dependent *M*‐*H* measurements by varying the angle of measurement *φ* from 0° to 90°. Figures S7 and S8 show the results for the as‐grown and treated FeB and FeBO (2% O_2_) heterostructures, respectively. Hysteresis loops measured along the in‐plane easy axis were selected as *φ* = 0 for both samples to better visualize the transition from in‐plane to out‐of‐plane contributions. In both systems, the magnetic easy axis remains predominantly in‐plane, although a weaker out‐of‐plane contribution is also present. Gating tends to reduce the out‐of‐plane contribution in terms of coercivity, while the squareness remains largely unchanged.

To disentangle the role of B in the magneto‐electric behavior of the heterostructures, time of flight‐energy (TOF‐E) elastic recoil detection analysis (ERD) was carried out to determine the composition across the samples.

Figure 2a,b shows the concentration profiles of Fe and B (at.%) as a function of depth for the as‐grown and voltage‐treated FeB and FeBO (2% O_2_) films gated for 60 min, respectively. Considering that the depth resolution uncertainty for B is below 2 nm for both FeB and FeBO (2% O_2_) (note that, for FeBO (2% O_2_), it is 20 TFU (thin film units) × 0.09 nm = 1.8 nm, see 4. Experimental section for further details), the shifts observed in FeB (Figure 2a) remain below 4 nm and therefore fall within the uncertainty range, indicating the absence of statistically significant B migration. In contrast, the FeBO (2% O_2_) film (Figure 2b) exhibits clear shifts of both Fe and B profiles toward deeper regions, evidencing a voltage‐induced transport of both Fe and B ions. The migration of both species toward the negatively biased Au electrode further indicates their cationic nature.

**FIGURE 2:**
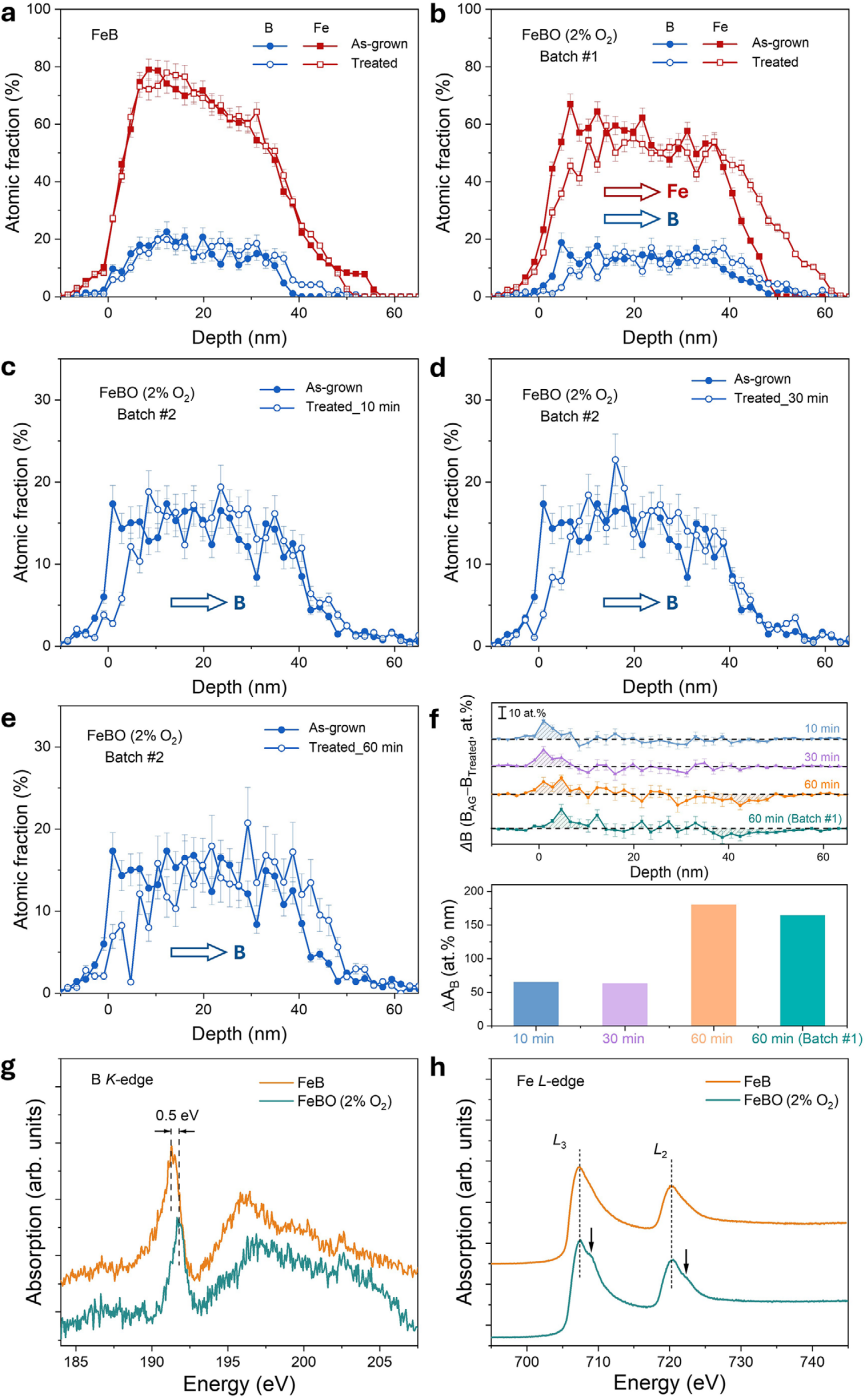
Compositional characterization of the heterostructures by TOF‐E ERD and XAS. (a) TOF‐E ERD depth profiles of Fe and B elements for the as‐grown and gated (−50 V for 1 h) FeB films. (b) TOF‐E ERD depth profiles of Fe and B elements for the as‐grown and gated (−50 V for 1 h) FeBO (2% O_2_) films, which correspond to the sample Batch #1. (c–e) are the TOF‐E ERD depth profiles of B for the FeBO (2% O_2_) films from a separate batch of samples prepared under identical growth conditions (Batch #2) gated at −50 V for 10, 30 and 60 min, respectively. The TOF‐E ERD depth profile of B for the as‐grown FeBO (2% O_2_) film is also plotted to serve as a reference. (f) Top: Difference between the ERD profiles of B of the as‐grown and gated states for the FeBO (2% O_2_) system. Bottom: absolute value of the area below and above the curves. (g) B *K*‐edge and (h) Fe *L*‐edge XAS spectra for the as‐grown FeB and FeBO (2% O_2_) films in fluorescence yield (FY) mode. The arrows in panel (b) indicate the direction of movement of Fe and B while gating.

To gain insight into the ion transport kinetics, additional TOF‐E ERD measurements were performed on FeBO (2% O_2_) films gated at −50 V for 10, 30, and 60 min (Figure 2c–e). For all actuation times, the B shifts clearly exceed the depth uncertainty limit, confirming their statistical significance and demonstrating voltage‐driven B migration. However, the migration distance does not scale monotonically with gating time, as the samples gated for 10 and 30 min exhibit comparable migration distances, whereas a marked enhancement is observed after 60 min. The ERD profiles for 10 and 30 min reveal migration mainly confined to the upper region of the film, suggesting that the early stages of actuation are dominated by local redistribution and short‐range migration processes near the electrolyte/film interface. In contrast, the sample gated for 60 min exhibits enhanced migration at the top region together with shifts at both interfaces, evidencing a more extensive redistribution of B throughout the film. It is worth noting that these duration‐dependent measurements were carried out on an independently prepared batch of samples grown under identical conditions to the FeBO (2% O_2_) sample gated for 60 min shown in Figure 2b. The excellent agreement between the resulting depth profiles confirms the reproducibility and robustness of the observed voltage‐driven B transport, with the independently measured sample displaying nearly identical redistribution behavior (Figure 2e). To facilitate visualization of these changes, Figure 2f presents the difference between the ERD profiles of the as‐grown and gated states. Although neither the migration distance nor the absolute integrated area evolves monotonically with gating time, all gated samples exhibit clear deviations from the as‐grown state, evidencing voltage‐driven B transport. The simultaneous appearance of B depletion and accumulation regions for the samples gated for 60 min further confirms the redistribution of B within the film upon actuation. Similarly to FeB, FeBO (5% O_2_) gated for 60 min (Figure S9) does not exhibit statistically significant changes in ion transport upon gating. The Fe profiles corresponding to 10 and 30 min do not show statistically significant changes within these time windows (not shown), suggesting that Fe migration requires longer actuation times to become appreciable.

Unfortunately, TOF‐E ERD cannot be used to determine the direction of voltage‐driven oxygen transport, since it cannot distinguish between oxygen incorporated from the atmosphere and oxygen that has undergone voltage‐induced movement. However, strong evidence for the upward movement of the O ions under voltage actuation can be observed from the permanent increase in *M*_S_, as shown in Figure 1g. This is ascribed to the voltage‐driven transport of ions toward the liquid electrolyte, partly reducing the FeBO to FeB. This permanent increase in *M*_S_ is not linked to changes in the B/Fe concentration along depth, since the B/(B+Fe) ratio remains nearly constant across the film (Figure S10). This behavior arises because Fe and B migrate in the same direction during gating, consistent with the cationic character of Fe ions and the cation‐like behavior of boron once it becomes electrochemically active. Therefore, the increase in *M*_S_ is associated with the partial reduction of FeBO toward FeB‐like environments. Additional evidence for the upward movement of the O ions upon voltage actuation can be detected in the HAADF‐STEM‐EELS characterization of the voltage‐treated sample, where it can be clearly observed that topmost oxide‐rich layer (i) doubles in thickness (from 5 nm in the as‐grown state, see Figure 1b, to 10 nm after voltage treatment) and (ii) loses Fe after gating (Figure 3).

**FIGURE 3:**
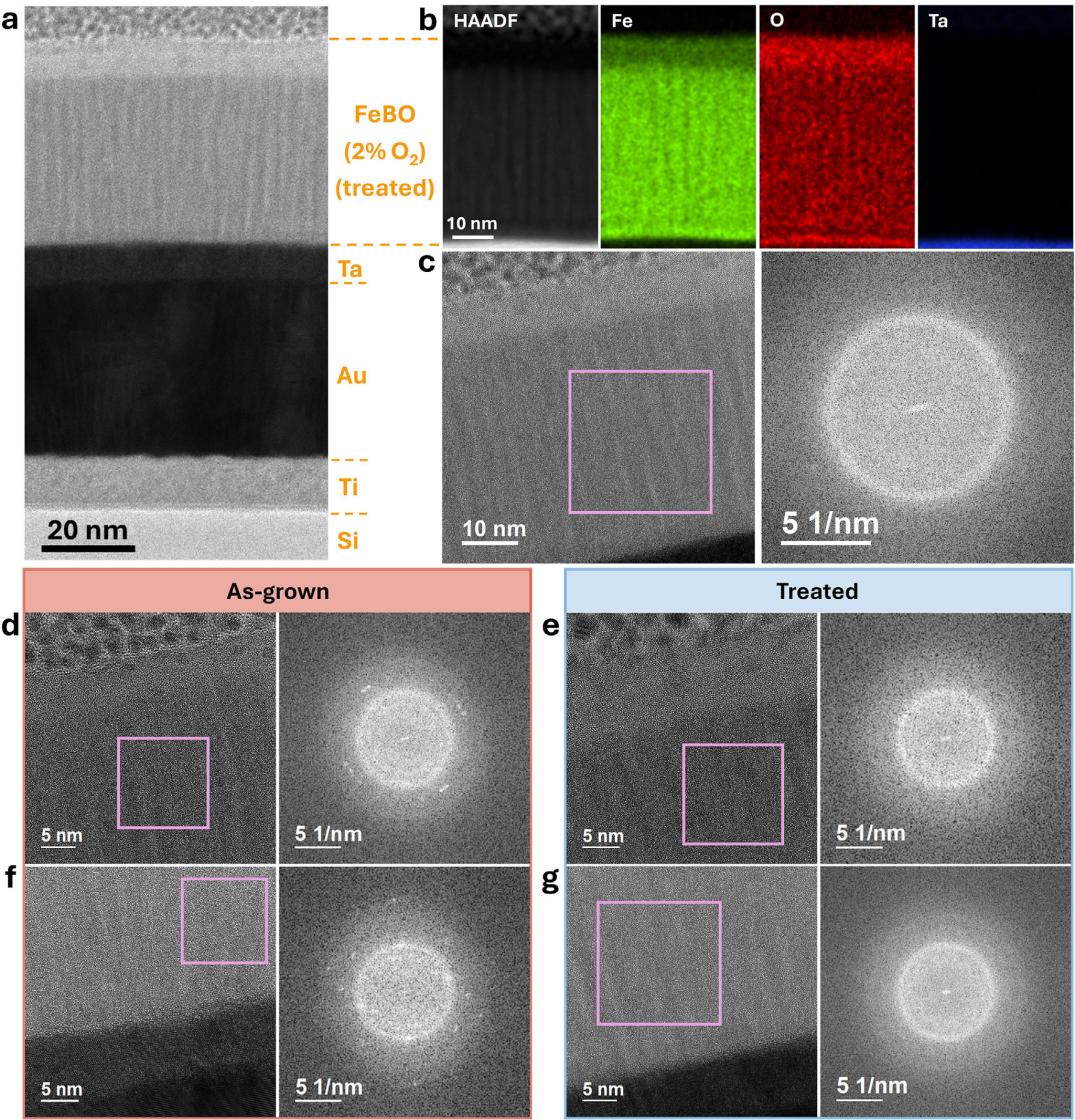
Microstructural and compositional characterizations of the as‐grown and voltage‐actuated FeBO (2% O_2_) films. (a) Cross‐sectional TEM image of the FeBO (2% O_2_) heterostructure after a gating actuation at −50 V for 1 h. (b) Representative HAADF‐STEM micrograph of the heterostructure and the corresponding Fe, O, and Ta elemental EELS mappings. (c) High‐resolution TEM image of the gated FeB‐O layer, together with the FFT pattern of the region enclosed by the red square. (d–g) Comparison of enlarged high‐resolution TEM images of the top (d, e) and bottom (f, g) regions of the heterostructures in their pristine (d, f) and gated (e, g) states. The corresponding FFT patterns for the regions marked with red squares are displayed in the right panels.

To elucidate the role of the oxidation state in the evolution of magneto‐electric properties, x‐ray absorption spectroscopy (XAS) was carried out in fluorescence yield mode (FY; sensitive to the bulk of the sample). Figure 2g,h shows the B *K* and Fe *L_2,3_* FY‐XAS spectra, respectively. As seen in Figure 2g, the peak located at around 191 eV of the B *K*‐edge XAS spectra shifts toward higher energies with increasing oxygen content, indicating a higher oxidation state of B [28]. Since the B ion movement is observed in FeBO (2% O_2_) but not in FeB, this suggests the existence of an oxidation‐state window that enables voltage‐driven B transport. This charge‐transfer effect also takes place in Fe, manifested as the newly developed shoulders at the higher energy ends of the Fe *L_2,3_* main peaks (indicated by the downward arrows in Figure 2h). Thus, the results indicate that, in the FeBO system, multi‐ion movement occurs within a finite oxidation‐state window of B, where the bonding configuration is sufficiently oxidized to activate mobility, yet not fully stabilized in a B_2_O_3_‐like state. Assigning absolute integer oxidation states to B based solely on B K‐edge XAS remains challenging because the spectral features are not exclusively determined by the oxidation state but are also strongly affected by the local coordination, bonding geometry, defects, crystal structure, and the pronounced covalent character of B‐containing systems [28, 29, 30]. Therefore, the B *K*‐edge chemical shift with respect to B_2_O_3_‐like bonding (|Δ*E*|) is used as a spectroscopic oxidation index to describe the B electronic state rather than to assign absolute oxidation states. Under this framework, B migration is activated within a finite oxidation‐state window corresponding to intermediate oxidation levels (|Δ*E*| ≈ 0.7 eV). Outside this window, either in the metallic‐like regime (FeB with |Δ*E*| ≈ 1.2 eV) or in the highly oxidized B_2_O_3_‐like regime (FeBO 5% O_2_ with |Δ*E*| ≈ 0 since the B K‐edge peak is close to that of the B_2_O_3_ reference), ionic transport is suppressed.

To further unravel the role of changes in the oxidation state in B, the magneto‐electric properties of the FeBO (5% O_2_) sample were also investigated. As shown in Figure S11, FeBO (5% O_2_) shows magneto‐ionics, evidencing clear electrochemical effects, where the heterostructure evolves from paramagnetic to ferromagnetic upon gating. This is linked to the higher oxygen content and the concomitant higher resistivity, which enhances electric field penetration [14]. As shown in Figure S12, for the as‐grown FeBO (5% O_2_) sample, the higher‐energy shoulder peaks observed in the FY‐XAS spectra of the FeBO (2% O_2_) sample become more intense, indicating an enhanced Fe oxidation state. Moreover, the peak in the B FY‐XAS spectrum of the as‐grown FeBO (5% O_2_) exhibits a considerable shift toward higher energies, approaching the energy of boron oxide (B_2_O_3_), strongly suggesting the formation of B_2_O_3_ when the samples are grown in large amounts of oxygen. The high stability of B_2_O_3_ [31] results in a system where B is less mobile compared to FeBO (2% O_2_), as confirmed by the reduced shifts of B concentration toward deeper parts upon gating in FeBO (5% O_2_) compared to FeBO (2% O_2_) (see the ERD results in Figure S9). However, minor residual contribution likely associated with partially oxidized or non‐ B_2_O_3_ local bonding environments cannot be ruled out. Thus, the results indicate that, in the FeBO system, to induce multi‐ion movement, an oxidation‐state window of B is required. However, the optimization of the oxidation states of the different ions to induce multi‐ion transport should depend on the ions involved and the material.

A remarkable feature induced by the multi‐ion movement in the FeBO (2% O_2_) upon gating is the observed increase in coercivity for most angles (Figure S5c). To gain insight into this effect, a detailed structural characterization was carried out using TEM and EELS (Figure 3). Figure 3a shows a cross‐sectional TEM image of the FeBO (2% O_2_) heterostructure after gating at −50 V for 1 h. The HAADF‐STEM image and Fe, O, and Ta EELS maps of the cross‐section confirm that the topmost layer is O‐rich but Fe‐depleted (Figure 3b), in agreement with the voltage‐driven transport of Fe ions downward toward the bottom electrode. Furthermore, the high‐resolution TEM image and FFT analysis in Figure 3c reveal that voltage application leads to an increased degree of nanostructuring of the films, with an amorphous‐like character. This is evidenced by the disappearance of diffraction spots, leaving only a diffuse diffraction ring. A closer examination of the high‐resolution images in Figure 3d–g, which compare the top and bottom regions of the microstructures along with their corresponding FFT patterns, confirms that this voltage‐induced structural modification occurs consistently throughout the films.

The coercivity of nanostructured systems typically decreases with reduced crystallinity (i.e., finer grain size) [32]. However, in amorphous‐like systems, increased structural disorder can promote magnetic inhomogeneities, local atomic disorder, and internal strains, which enhance domain‐wall pinning and magnetic anisotropy gradients, ultimately leading to higher coercivities [33, 34, 35]. In such systems, the absence of long‐range crystallographic order suppresses conventional magnetocrystalline anisotropy, while spatial fluctuations in local atomic environments and exchange interactions create an inhomogeneous magnetic energy landscape that hinders magnetization reversal [35]. To better understand the origin of this coercivity increase, structural characterization at the atomic level was performed by variable‐energy positron annihilation lifetime spectroscopy (VEPALS, Figure 4 and Note S1). In FeBO (2% O_2_) heterostructures, small vacancy‐clusters (i.e., τ_1_) are the majority defect type, with a relative intensity (i.e., *I*_1_) exceeding 55% (Figure 4a,b). The size of vacancy agglomerations is larger in the sub‐surface region, decreasing toward the buffer layer, which is likely related to the oxygen overstoichiometry and Fe depletion, suggesting the presence of iron vacancies. The second lifetime component τ_2_ can be associated with open volumes at the grain boundaries and their intersections since its counterpart is smaller compared to τ_1_. Interestingly, the defect structure in the buried regions of FeBO (2% O_2_) is modified by voltage actuation. Specifically, *I*_1_ increases by more than 5% after voltage actuation, indicating a higher density of small vacancy clusters. This increase in defect density likely promotes stronger local atomic disorder and strain fluctuations, enhancing magnetic anisotropy gradients and domain‐wall pinning, which ultimately contributes to the observed increase in coercivity [33, 34, 35]. At the same time, a slight thickening of the defected sub‐surface region is found in the relative intensity depth profile, in agreement with the TEM investigations (Figure 3a,b).

**FIGURE 4:**
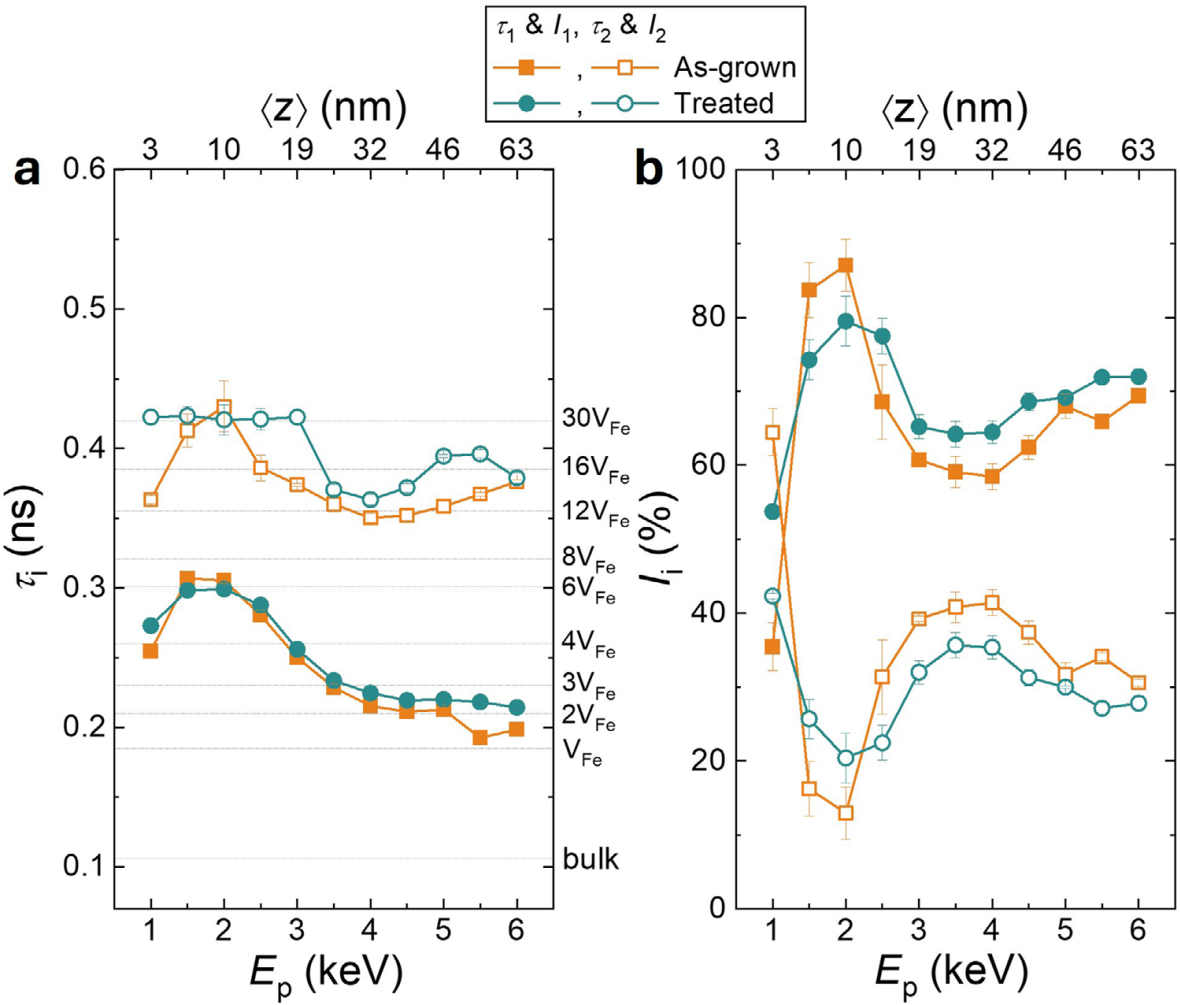
Defect characterization of the FeBO (2% O_2_) films in both pristine and voltage‐actuated states by VEPALS. (a) Positron lifetime components (τ_1_ and τ_2_), and (b) the corresponding relative intensities (*I*_1_ and *I*_2_), as a function of positron mean implantation depth (〈*z*〉) and implantation energy (*E*_p_) (shown in the top and bottom axes, respectively) for the as‐grown and voltage‐treated FeBO (2% O_2_) films. See Note S1 for further details on the correlation of positron lifetime with the number of Fe vacancies (V_Fe_).

## 3 Conclusion

Our findings reveal a novel voltage‐driven multi‐ion (B, Fe, and O) transport in an FeBO system. As O content increases, the system transitions from an electrostatic‐like (weakly electrochemical) response to a more pronounced electrochemical (magneto‐ionic) behavior, driven by an increase in electrical resistivity. The voltage‐driven transport of B, demonstrated for the first time, is enabled and triggered by tuning its oxidation state through oxygen incorporation. B magneto‐ionics becomes activated within a specific oxygen concentration range. Lower oxygen levels result in weakly magneto‐ionic systems with low resistivity, while higher oxygen concentrations lead to the formation of stable boron oxide, limiting voltage‐driven B transport. In addition, the voltage‐induced multi‐ion movement leads to a remarkable coercivity increase, which is related to the increased degree of amorphization in voltage‐treated samples. These findings provide a pathway for designing multi‐ion manipulation through charge‐transfer engineering, leveraging elements with dissimilar electron affinities, and enabling the integration of multiple functionalities and on‐demand programmable magneto‐ionics. Moreover, expanding the range of mobile species to include B broadens the scope of magneto‐ionic applications, particularly in fields where boron plays a crucial role, such as electronics [36] or nuclear therapies [37] (e.g., boron neutron capture therapy).

## 4 Experimental Section

### 4.1 Sample Preparation

50 nm‐thick FeBO films with varying oxygen content were grown by DC magnetron sputtering using an Fe_80_B_20_ (nominal atomic %) target on Si(100) wafers, previously coated with 15 nm Ti, 50 nm Au, and 10 nm Ta. All depositions were carried out at room temperature using an AJA International ATC 2000‐V Sputtering System with a base pressure < 1 × 10^−7^ Torr. The oxygen content was changed by varying the O_2_/Ar flow rate (i.e., $\frac{O_{2}\;flow}{O_{2}\;flow+\mathrm{Ar}\;flow}$) in the sputtering chamber: 0 (no oxygen), 2, and 5%; denoted as FeB, FeBO (2% O_2_), and FeBO (5% O_2_), respectively. As a standard procedure, the target was pre‐sputter cleaned at a power of 100 W, whereas the sputter deposition of the films was performed at 55 W. The total pressure is fixed at 3 × 10^−3^ Torr for the growth. The substrate‐to‐target distance was approximately 10 cm. Prior to the growth of the Ta buffer layer and FeB(O) films, the Au layer was partially masked to serve as the working electrode for subsequent magneto‐electric characterization.

### 4.2 Magneto‐Electric Characterization

Magnetic and magneto‐electric measurements were carried out at room temperature using a vibrating sample magnetometer from MicroSense (LOT, Quantum Design). The magnetic fields were applied along the film plane direction. For standard hysteresis loops, the maximum applied field was 20 kOe. Magneto‐electric measurements were carried out while applying voltages, using an external B2902A power source, between the counter electrode (a Pt wire) and the Au working electrode in a custom‐made electrolytic cell [5, 8], filled with liquid electrolyte: anhydrous propylene carbonate solvating Na^+^ and OH^–^ species (10 – 25 ppm). The magnetization was determined by normalizing the magnetic moment to the sample volume of the as‐grown film exposed to the electrolyte. Any diamagnetic and paramagnetic contributions from the hysteresis loops were subtracted by correcting the linear slopes at high fields.

### 4.3 Structural and Compositional Characterization

High‐resolution transmission electron microscopy (TEM), high‐angle annular dark‐field scanning transmission electron microscopy (HAADF‐STEM), and electron energy loss spectroscopy (EELS) were carried out on a Spectra 300 (S)TEM microscope (Thermo Fisher Scientific), operated at 200 kV. Prior to TEM observations, cross‐sectional lamellae were prepared by focused ion beam, placed onto a copper transmission electron microscopy grid, and sputter‐coated with a protective platinum layer.

Grazing‐incidence X‐ray diffraction measurements of the as‐grown FeB and FeBO (2% O_2_) films were recorded using Cu K_α_ radiation at an incidence angle of 1^0^.

TOF‐E ERD [38] was carried out to determine the Fe, B, and O concentrations along depth. An impinging ion beam of 10.8 MeV ^79^Br^5+^ particles was used. The sample was tilted to a grazing incidence angle of 75° between the ion beam and the normal direction of the film surface. Elastic collisions between the incident ions and target atoms generate recoiling species, whose mass, energy, and forward‐emission yield were measured using a multi‐dispersive detector telescope positioned at 40° relative to the beam direction. ERD provides quantitative elemental depth profiles by probing atomic areal densities; therefore, the original results are obtained in units of 1 × 10^15^ atom/cm^2^ (thin film units, TFU). The depth profile is derived from the energy loss of ions in matter [39].

To facilitate a more direct comparison between the depth of ionic motion and results obtained from complementary techniques, such as EELS concentration maps, the ERD depth scales were converted from areal density units (TFU) into length units (nm). This conversion requires knowledge of the atomic density of the material, which was estimated by combining the film thickness obtained from TEM with the total areal density determined by ERD. For the FeBO (2% O_2_) sample, TEM measurements (Figure 3) indicate a film thickness of approximately 50 nm, while ERD yields a total areal density *N_A_* of around 530×10^15^ at./cm^2^. These values provide an atomic density ρ_*A*_ (*N_A_* divided by the thickness) of approximately 1.1 × 10^23^ at./cm^3^. The depth conversion was then performed according to:

$$\mathrm{Depth}\;(\mathrm{nm})\;=\frac{N_{A}}{\rho_{A}}\;\times 10^{7}$$

where *N_A_* is the areal density (at./cm^2^), ρ_*A*_ is the atomic density (at./cm^3^), and the factor 10^7^ converts cm into nm. Accordingly, 1 TFU (1 × 10^15^ at./cm^2^) corresponds to approximately 0.09 nm for the FeBO (2% O_2_) film. The same procedure was subsequently applied to all ERD depth profiles.

The concentration uncertainties were estimated from the counting statistics of the ERD depth profiles. The total counts obtained from the cut files for B and Fe were first distributed over the number of depth intervals used to construct the depth profiles, where the plateau width was defined from the full width at half maximum. The statistical uncertainty, σ_*N*_, associated with each depth point was then calculated assuming Poisson counting statistics, i.e., $\sigma_{N}=\sqrt{N}\;$, where *N* is the number of counts assigned to a given depth interval. In counting experiments such as ERD, σ_*N*_ represents both the standard deviation and the statistical uncertainty of the measured counts. Since the elemental concentration *C* at each depth is obtained by normalizing the elemental counts to the total signal and converting them into atomic concentration, the count uncertainty was propagated through this normalization procedure. Consequently, the concentration uncertainty is $\sigma_{C}=\left(C\sqrt{N}\right)/N$, leading to larger relative uncertainties for depth regions with lower statistics and smaller uncertainties in the plateau regions with higher counts. These propagated uncertainties were used as the concentration error bars shown in the depth profiles.

The depth uncertainty was estimated from the near‐surface depth resolution in the first approximation because, in ERD measurements, the depth resolution decreases with increasing depth due to energy straggling and multiple scattering of ions within the material. The quantification of the depth uncertainty presented below is carried out for B; the same analysis applies analogously to Fe.

The surface depth resolution, *u_d_*, is calculated as *u_d_* = *u_E_* /|*S*|_*p*_, where *u_E_* is the energy resolution of the detected recoils and |*S*|_*p*_ is the projected stopping cross section accounting for the energy losses of both the incoming primary ions and the outgoing recoils along the experimental geometry, given by:

$${\left|S\right|}_{p}=k_{r}\;\frac{S_{p}\left(E_{Br}\right)}{\sin\alpha }+\frac{S_{r}\left(E_{B}\right)}{\sin\beta }$$

where *k_r_* is the recoil kinematic factor, *S_p_* (*E_Br_*) is the stopping power of the incident Br ions (12.6 MeV), *S_r_* (*E*_B_) is the stopping power of the recoiled B atoms (≈ 3 MeV), and α and β are the incidence and recoil angles, respectively. The stopping powers were obtained from the SIMNRA software package (Simulation of Backscattering Spectra), which calculates ion stopping and scattering processes for ion beam analysis techniques using sample‐specific compositions [40]. The energy resolution for ≈3 MeV B recoils is then *u_E_* ≈ 28 keV based on the performance of an identical TOF‐E ERD setup as the one used in this work [41], resulting in near‐surface boron depth resolutions of ≈20 TFU, where 1 TFU is 1 × 10^15^ at./cm^2^.

X‐ray absorption spectroscopy (XAS) at the B *K* and Fe *L*_2_,_3_ edges was carried out at the BL29‐BOREAS beamline located at the ALBA Synchrotron [42], under ultra‐high vacuum conditions (base pressure ∼10^−10^ Torr). The spectra were collected at room temperature in fluorescence yield (FY) detection mode, with the incoming x‐ray beam direction forming an angle of 15 degrees relative to the sample normal vector. The Fe *L*_2,3_ edge XAS spectra were obtained from averaging 8 consecutive scans, whereas the B *K* edge XAS data represent averages of 20 scans, to ensure quality datasets and to minimize any possible beam‐related spectral shifts.

Defect characterization was carried out by variable‐energy positron annihilation lifetime spectroscopy (VEPALS). VEPALS measurements were conducted at the Mono‐energetic Positron Source (MePS) beamline at Helmholtz–Zentrum Dresden – Rossendorf (Germany) [43]. A CeBr_3_ scintillator detector together with a Hamamatsu R13089‐100 photomultiplier tube for the gamma photon detection was employed. A Teledyne SPDevices ADQ14DC‐2X digitizer with a 14‐bit vertical resolution and 2GS/s (gigasamples per second) horizontal resolution was utilized for the processing of signals. The overall time resolution of the measurement system is ≈ 0.250 ns, and all spectra contain at least 1×10^7^ counts. A typical lifetime spectrum *N*(*t*), which is the absolute value of the time derivative of the positron decay spectrum, is described by $N\;\left(t\right)=\;R\left(t\right)*\sum_{i\;=\;1}^{k+1}\frac{I_{i}}{\tau_{i}}e^{-t/\tau_{i}}+\mathrm{Background}$, where k is the number of different defect types contributing to the positron trapping, which are related to k + 1 components in the spectra with individual lifetimes τ_i_ and intensities *I*_i_ (∑*I*_i_ =  1). The instrument resolution function *R*(*t*) is a sum of two Gaussian functions with distinct intensities and relative shifts, both depending on the positron implantation energy, *E*_P_. It was determined by measuring a reference sample, i.e. yttria‐stabilized zirconia, which exhibits a known single lifetime component of 182 ± 3 ps. The background was negligible. All the spectra were deconvoluted using a non‐linear least‐squares fitting method, minimized by the Levenberg‐Marquardt algorithm in the software package PALSfit [44], into 2 major lifetime components, which directly evidence localized annihilation at 2 different defect types (sizes; τ_1_ and τ_2_). The shortest lifetime component τ_1_ represents smaller vacancy‐clusters, while the lifetime component τ_2_ accounts for larger vacancy‐clusters linked to grain boundaries or small pores. The relative intensity (*I*_i_) of each component can be regarded to some extent as the concentration of each defect type [45]. In general, positron lifetimes may be overestimated in the topmost regions of films due to surface roughness and broken symmetry, but these effects are reduced beyond depths of 20 nm (>2 keV in terms of positron implantation energy *E*_P_, see below). Therefore, at depths exceeding 20 nm (>2 keV), positron lifetimes can be considered fully representative of the bulk film properties. The positron lifetime and its intensity have been probed as a function of positron implantation energy *E*_P_, which is related to the mean implantation depth 〈*z*〉 following: $\left\langle z\right\rangle \left(\mathrm{nm}\right)=\frac{23.9}{\rho (\frac{g}{cm^{3}})}\;E_{P}{\left(\mathrm{keV}\right)}^{1.69}$ [46]. 〈*z*〉 is an approximate measurement of depth since it does not account for positron diffusion.

### 4.4 Electrical Characterization

To assess the electrical properties, 50‐nm FeB(O) films were deposited directly onto high‐resistive SiO_2_/Si substrates, following the same sputtering conditions used for depositing magneto‐ionic heterostructures. The electrical transport measurements were carried out at room temperature using the van der Pauw configuration. For as‐grown FeB, FeBO (2% O_2_), and FeBO (5% O_2_) films, resistivity values of 2.0 × 10^−4^, 2.7 × 10^−4^, and 1.7 ohm cm are obtained, respectively.

## Author Contributions

Z.M. and E.M. conceived the original idea and planned the experiments. E.M. led the investigation. Z.M., E.P, and S.P. designed and synthesized the FeB and FeBO heterostructures. Z.M., H.T., A.A.‐L., D.M.‐B., and A.Q. carried out the magnetic and magneto‐electric measurements and analyzed the data. M.O.L., E.H., and A.W. performed the PALS characterization and analyzed the data. J.H.‐M. carried out the XAS characterization and analyzed the data. K.‐A.K. and J.M. performed the TOF‐E ERD measurements and analyzed the data. A.Q., J.N., and J.S. carried out the GIXRD, TEM, and STEM characterization. The article was written by Z.M. and E.M. with contributions from all co‐authors.

## Conflicts of Interest

The authors declare no conflicts of interest.

## Supporting information




**Supporting File**: advs76552‐sup‐0001‐SuppMat.docx.

## Data Availability

The data that support the findings of this study are available from the corresponding author upon reasonable request.
