# Supplementary material for: Charge‐Transfer‐Mediated Boron Magneto‐Ionics: Towards Voltage‐Driven Multi‐Ion Transport
Source: Adv Sci (Weinh). 2026 Jul 11;13(55):e76552. doi: 10.1002/advs.76552 (PMC13355926; doi:10.1002/advs.76552)
Supplement: Supplementary file 1 [file ADVS-13-e76552-s001.docx]

Supporting Information

Charge-transfer-mediated boron magneto-ionics: Towards voltage-driven multi-ion transport

Zheng Ma,* Karim-Alexandros Kantre, Huan Tan, Aitor Arredondo-López, Maciej O. Liedke, Javier Herrero-Martín, Eric Hirschmann, Andreas Wagner, Daniel Mora-Blanco, Alberto Quintana, Salvador Pané, Eva Pellicer, Josep Nogués, Johan Meersschaut, Jordi Sort*, Enric Menéndez*

**Outline**

- Supplementary Figure S1: The influence of oxygen on the microstructure of FeBO films.

- Supplementary Figure S2: Further TEM characterization of as-grown FeBO (2% O_2_) films.

- Supplementary Figure S3: Grazing-incidence X-ray diffraction measurements of the as-grown FeB and FeBO (2% O_2_) films.

- Supplementary Figure S4: In-plane angular-dependent $M$-$H$ measurements for the FeB films in the as-grown and treated states.

- Supplementary Figure S5: In-plane angular-dependent $M$-$H$ measurements for the FeBO (2% O_2_) films in the as-grown and treated states.

- Supplementary Figure S6: Magnetic hysteresis loops of the as-grown and gated FeO (2% O_2_) films.

- Supplementary Figure S7: In-plane to out-of-plane angular-dependent $M$-$H$ measurements for the FeB films in the as-grown and treated states.

- Supplementary Figure S8: In-plane to out-of-plane angular-dependent $M$-$H$ measurements for the FeBO (2% O_2_) films in the as-grown and treated states.

- Supplementary Figure S9: Fe and B depth profiles by TOF-E ERD of the as-grown and gated FeBO (5% O_2_) films.

- Supplementary Figure S10: Depth-dependence of the relative atomic percentage of B with respect to the B and Fe atomic percentages of the as-grown and gated FeB and FeBO (2% O_2_) films.

- Supplementary Figure S11: Magnetic hysteresis loops of the as-grown and gated FeBO (5% O_2_) films.

- Supplementary Figure S11: FY-XAS spectra of as-grown FeB, FeBO (2% O_2_), and FeBO (5% O_2_) films.

- Supplementary Note 1: Correlation between positron lifetime and the number of Fe vacancies ($V_{Fe}$).

- Supplementary References

**Supplementary Figure S1:** The influence of oxygen on the microstructure of FeBO films

**Figure S1.** Representative TEM micrographs of the cross-sections of as-grown a) FeB, and b) FeBO (2% O_2_) films, both are 50 nm in thickness, showing the feature of columnar growth when the heterostructures are sputtered in an oxidized atmosphere.

**Supplementary Figure S2:** Further TEM characterization of as-grown FeBO (2% O_2_) films

**Figure S2.** Further TEM characterization of as-grown FeBO (2% O_2_) films. Crystallites compatible with Fe can be observed in certain regions as shown in the enlarged view of the marked yellow square (PDF^®^ 00-001-1262).

**Supplementary Figure S3:** Grazing-incidence X-ray diffraction measurements of the as-grown FeB and FeBO (2% O_2_) films.

**Figure S3.** Grazing-incidence X-ray diffraction measurements of the as-grown FeB and FeBO (2% O_2_) films using Cu K_α_ radiation at an incidence angle of 1⁰. For phase identification, Materials Project database has been used.^[1]^

**Supplementary Figure S4:** In-plane angular-dependent $M$-$H$ measurements for the FeB films in the as-grown and treated states.

**Figure S4.** In-plane hysteresis loops measured at selected angles of *θ* = 0⁰, *θ* = 30⁰ (magnetic hard axis), and *θ* = 105⁰ (magnetic easy axis) for a) as-grown and b) treated FeB films. Panels c) and d) show the full in-plane angular dependence of the coercivity and squareness, respectively. Note that the samples have an approximately rectangular shape with active area (*i.e.*, excluding the Au working electrode) dimensions of ≈ 5 mm × 8 mm. Note that 0° corresponds to VSM measurements performed with the magnetic field applied along the shortest dimension of the sample.

**Supplementary Figure S5:** In-plane angular-dependent $M$-$H$ measurements for the FeBO (2% O_2_) films in the as-grown and treated states.

**Figure S5.** In-plane hysteresis loops measured at selected angles of *θ* = 0⁰, *θ* = 105⁰, and *θ* = 165⁰ for a) as-grown and b) treated FeBO (2% O_2_) films. Panels c) and d) show the full in-plane angular dependence of the coercivity and squareness, respectively. Note that the samples have an approximately rectangular shape with active area (*i.e.*, excluding the Au working electrode) dimensions of ≈ 5 mm × 8 mm. Note that 0° corresponds to VSM measurements performed with the magnetic field applied along the shortest dimension of the sample.

**Supplementary Figure S6:** Magnetic hysteresis loops of the as-grown and gated FeO (2% O_2_) films.

**Figure S6.** Hysteresis loops of the as-grown and gated (–50 V for 1 h) FeO (2% O_2_) films. The inset presents a zoom-in of the high-field region marked by the dashed rectangle. Note the slight change (≈ 2%) change in $M_{S}$ in the voltage-treated sample in the return branch of the loop.

**Supplementary Figure S7:** In-plane to out-of-plane angular-dependent $M$-$H$ measurements for the FeB films in the as-grown and treated states.

**Figure S7.** In-plane to out-of-plane hysteresis loops measured at selected angles of *ϕ* = 0⁰, *ϕ* = 15⁰, *ϕ* = 30⁰, *ϕ* = 45⁰, *ϕ* = 75⁰, and *ϕ* = 90⁰ for a) as-grown and b) treated FeB films. Panels c) and d) show the full in-plane to out-of-plane angular dependence of the coercivity and squareness, respectively. Note that hysteresis loops showing in-plane easy axis behavior were selected as *ϕ* = 0⁰ with the goal to better visualize possible changes in magnetic anisotropy associated with the transition from in-plane to out-of-plane contributions.

**Supplementary Figure S8:** In-plane to out-of-plane angular-dependent $M$-$H$ measurements for the FeBO (2% O_2_) films in the as-grown and treated states.

**Figure S8.** In-plane to out-of-plane hysteresis loops measured at selected angles of *ϕ* = 0⁰, *ϕ* = 15⁰, *ϕ* = 30⁰, *ϕ* = 45⁰, *ϕ* = 75⁰, and *ϕ* = 90⁰ for a) as-grown and b) treated FeBO (2% O_2_) films. Panels c) and d) show the full in-plane to out-of-plane angular dependence of the coercivity and squareness, respectively. Note that hysteresis loops showing in-plane easy axis behavior were selected as *ϕ* = 0⁰ with the goal to better visualize possible changes in magnetic anisotropy associated with the transition from in-plane to out-of-plane contributions.

**Supplementary Figure 9:** Fe and B depth profiles by TOF-E ERD of the as-grown and gated FeBO (5% O_2_) films.

**Figure S9.** Fe and B depth profiles by TOF-E ERD for the as-grown and gated FeBO (5% O_2_) films.

**Supplementary Figure S10:** Depth-dependence of the relative atomic percentage of B with respect to the B and Fe atomic percentages of the as-grown and gated FeB and FeBO (2% O_2_) films.

**Figure S10.** Evolution of the atomic percentage of B (% of B/(B+Fe)) as a function of depth for FeB and FeBO (2% O_2_) films, shown in the left and right panels, respectively. Note that the B/Fe ratio does not significantly change in depth, in agreement with a dual-cation movement of Fe and B ions during the electrolyte gating process.

**Supplementary Figure S11:** Magnetic hysteresis loops of the as-grown and gated FeBO (5% O_2_) films.

**Figure S11.** Hysteresis loops of the as-grown and gated (–50 V for 1 h) FeBO (5% O_2_) films. Upon gating, consecutive hysteresis loops were recorded until a permanent ferromagnetic response is reached.

**Supplementary Figure S12:** FY-XAS spectra of as-grown FeB, FeBO (2% O_2_), and FeBO (5% O_2_) films.

**Figure S12.** Fluorescence yield (FY) XAS spectra at the a) B *K*-edge, and b) Fe *L*-edge for the as-grown FeB, FeBO (2% O_2_), and FeBO (5% O_2_) films. The bottom panel of a) presents the B_2_O_3_ reference spectrum (peaked at 192.2 eV), collected from a pyrex borosilicate glass. The gradual shift of the B *K*-edge peak indicates the increased B oxidation. The occurrence of the shoulder peaks at higher energy ends in FeBO (2% O_2_) implies a slight increase in Fe oxidation. In the FeBO (5% O_2_) film, the shoulder peaks become stronger, indicating much enhanced oxidation state.

**Supplementary Note 1:** Correlation between positron lifetime and the number of Fe vacancies ($V_{\mathrm{Fe}}$)

For as-grown and voltage-treated FeB and FeBO (2% O_2_) heterostructures, $\tau_{1}$ values correspond to vacancy-clusters ranging from 3 to 8 Fe vacancies ($V_{\mathrm{Fe}}$ in Figure 4). With respect to $\tau_{2}$, which represents larger vacancy-clusters, clusters larger than 16 Fe vacancies ($V_{\mathrm{Fe}}$ in Figure 4) are observed in both as-grown and voltage-treated FeB and FeBO (2% O_2_) samples.^[2]^ To use Fe as an analogue here is justified as evidenced by the TEM FFT analysis indicating that the phase is close to metallic Fe (Figure 1c).

**Supplementary References**

[1] A. Jain, S. P. Ong, G. Hautier, W. Chen, W. D. Richards, S. Dacek, S. Cholia, D. Gunter, D. Skinner, G. Ceder, K. A. Persson, *APL Mater.* **2013**, 1, 011002.

[2] J. Čížek, O. Melikhova, Z. Barnovská, I. Procházka, R. K. Islamgaliev, *J. Phys. Conf. Ser.* **2013**, 443, 012008.
